# Remembering Social Events: A Construal Level Approach

**DOI:** 10.1177/01461672211038188

**Published:** 2021-08-18

**Authors:** Natalie A. Wyer, Timothy J. Hollins, Sabine Pahl

**Affiliations:** 1 University of East Anglia, Norwich, UK; 2 University of Plymouth, UK; 3 University of Vienna, Austria

**Keywords:** memory, meta-analysis, construal level

## Abstract

Social events are rich in information, yet research into how people remember such events has typically been limited to considering one aspect (e.g., faces, behaviors) at a time. Based on an internal meta-analysis of a program work encompassing 15 laboratory, field, and on-line experiments involving 1,230 participants, we found that construal level influences both the ability to recognize people involved in the event (*d* = 0.30) and the way the social aspects of the event are described (average *d* = 0.48). In contrast, memory for background objects/scenes that were present during the event was unaffected by construal level. We discuss these findings in terms of their implications for both event memory (and situations where different aspects of the same event are remembered) and for construal level (and the question of how and when construal is likely to affect memory).

Social encounters are rich in information—not only do they involve other people whose appearance and behavior are easily observed and encoded in terms of their social group or trait implications, but they often take place in complex physical environments. Moreover, all of these aspects are dynamic—others’ behavior and its meaning, as well as one’s view of (and attention to) the physical environment, may change frequently over time, often within a single interaction. All of this information may be attended to, encoded, and eventually stored in memory, available to be retrieved at a later point in time to reconstruct what happened during the encounter. Although many aspects of how people retrieve information from event representations have been studied empirically (for a review of research on memory for behaviors, see Hamilton & Sherman, 1996; N. A. Wyer, 2013; for a review of research on memory for faces, see Bruce & Young, 1986, 2012; and for a review of research on memory for objects and natural scenes, see Hollingworth, 2006; Hollingworth et al., 2001), each of these investigations has focused on memory for a single aspect of an event. In the present article, we report a program of work that sought to explore how people remember a wide spectrum of social and non-social information to which they might be exposed during a social encounter.

In doing so, we were particularly interested in discovering how information about different elements of an event can be retrieved using different processing styles. As reviewed in the following, there are potential parallels between modes of processing as applied to visual and behavioral information. Not only can visual stimuli (e.g., faces) be processed in terms of both their specific features and their holistic appearance, but specific behavior observations can also be construed in terms of both their specific details and their broader meaning (Winter & Uleman, 1984). To further our understanding of whether such distinctions share common processes, we adopted the framework of Construal Level Theory (CLT; Liberman & Trope, 2014; Trope & Liberman, 2010).

Construal Level theory (Liberman & Trope, 2014; Trope & Liberman, 2010) focuses on how events that are psychologically close or distant are interpreted, or construed. In particular, it posits that thinking about events that are close (e.g., in time or space) induces a “low-level construal,” characterized by more concrete or detailed processing related to the event. In contrast, thinking about events that are farther away induces a “high-level construal,” characterized by more abstract or holistic processing. For example, when planning what to pack for a holiday the weekend before leaving, one is likely to think about specific items (flip flops, toothbrush, paperback novel). However, when thinking about what to bring for the same holiday several months in advance, one is more likely to consider broad categories of items (clothing, toiletries, entertainment).

In the present work, we explored the possibility that the distinction between low-level and high-level construal might have parallel effects on visual and semantic processing of a social event: More specifically, we tested the hypothesis that high-level (vs low-level) construal would lead to more *holistic* (vs. *featural*) processing of faces encountered during an event, as well as more *abstract* (vs. *concrete*) processing of behaviors executed during the encounter. As a result, we should expect that faces and behavioral information should be remembered in different ways depending on the level of construal at which one is operating. Because attention during social events tends to focus on *people* (the participants in the event) to the neglect of background information (e.g., objects; Wentura et al., 2000), we anticipated that construal level would have a stronger influence on how people remember social information, in comparison to background or contextual information. Below, we review existing evidence for these hypotheses and consider possible mechanisms that might underlie the effects of construal level on social memory.

## How Do Psychological Distance and Construal Level Affect Processing Style?

Manipulations of various dimensions of psychological distance have been found to influence subsequent decisions and judgments relating to that target event (e.g., Liberman & Trope, 1998; Liberman, Trope, McCrea & Sherman, 2007) as well as to processing on unrelated tasks (e.g., Fujita, Trope, et al., 2006; Smith & Trope, 2006). Notably, manipulations of psychological distance (versus proximity) have been reported to improve performance on tasks believed to measure global processing, such as the Snowy Pictures Test and the Gestalt Completion Test (Wakslak et al., 2006) while impairing performance on tasks believed to measure local processing such as the Embedded Figures Test (Smith & Trope, 2006; Witkin et al., 1971). Thus, psychological distance (and by extension construal level) appears to have broad implications for information processing styles, which can then be transferred to subsequent logically unrelated tasks.

The link between psychological distance and construal level is believed to derive from different attentional processes applied to proximal vs. distal events. The more immediate or proximal an action or event is, the more it necessitates attention to the steps involved in carrying it out. For example, when standing at your front door and reaching for your house keys, attention is guided toward the motor responses required to fit the key in the lock to open the door. However, while at work in the mid-afternoon and thinking about arriving home at the end of the day, your attention is more likely focused on the goal to start the evening in the comfort of your own home—fitting the key to the front door lock is but one detail of the event and is unlikely to attract much attention. Thus, proximal events focus our attention on *how* to do things, whereas distal events focus our attention on *why* we do things.

Supporting this reasoning, construal level has also been manipulated without reference to any dimension of distance, but asking participants to describe “how” vs. “why” they would undertake an action (e.g., maintaining good health). As suggested above, the question of “how” has been found to induce low-level construal, while the question of “why” they would undertake the same action induces high-level construal (Freitas et al., 2004). Such exercises are intended to be more direct manipulations of construal level, as they require either concrete and detailed thinking (to describe how something is done) or more abstract and holistic thinking (to describe why something is done). Such manipulations have demonstrably affected not only processing of the target action but also processing that occurs in subsequent tasks (e.g., Fujita, Trope, Libeman, & Levin-Sagi, 2006).

Thus, CLT has gained influence in recent years for its ability to explain and predict a wide range of outcomes, from perception (Liberman & Förster, 2009) to judgments and decisions (Liberman, & Wakslak, 2007). Yet to date, tests of CLT within a single study have largely focused on specific and isolated outcomes. As outlined in the following, the case of event memory provides an opportunity to test the implications of CLT for memory across a broad range of stimuli (visual vs. behavioral), aspects of those stimuli (featural vs. holistic), contexts (laboratory vs. in the field vs. in one’s personal past), and physical involvement (being physically present during the event vs. observing a recording of a past event).

## Information Available Within Social Events

Our key assertion is that the information contained within a social event can be encoded and retrieved using distinct modes of cognitive processing. Construal Level Theory (Liberman & Trope, 2014; Trope & Liberman, 2010) posits that differences in the way a stimulus is construed have general implications for concurrent and subsequent information processing. Specifically, “low-level” construal involves interpreting a stimulus in a concrete manner, and lends itself to more detail-oriented or feature-focused processing. In contrast, “high-level” construal entails interpreting a stimulus in a more abstract manner and results in more holistic, gestalt, or configural processing. Although research stemming from Construal Level Theory has typically examined consequences for processing the very object of construal, preliminary evidence (discussed below) suggests that construal manipulations also carry over to influence processing style in relation to unrelated stimuli. In this section, we briefly review how relatively abstract vs. detail-oriented processing (argued to characterize high-level vs low-level construal, respectively) influences memory for different elements of social events.^1^

## Recognizing Faces

An extensive literature of face recognition supports the proposal that accurate face recognition is enhanced when people adopt a global or holistic processing style and hindered when they adopt a featural processing style. This support comes from two lines of research. In the first of these, researchers have manipulated processing style after encoding, but prior to test, and observed differences in performance on face recognition tasks. For example, Macrae and Lewis (2002) reported that participants who completed the “global” version of a Navon task (in which they identified large letter shapes; see Navon, 1977) during the retention interval were nearly three times more likely to correctly identify (from a line-up) a perpetrator of a crime than were participants who completed the “local” version of the same task (in which they identified smaller letters that formed the large letter shape). This effect has since been replicated in field studies (Perfect et al., 2007) and suggests that global processing of Navon letters carries over to influence face processing in an unrelated task. Moreover, Weston and Perfect (2005; see also Perfect, 2003) found that participants who first completed a local Navon task were quicker at recognizing one half of a composite face (i.e., the top of one face paired with the bottom of a different face). In other words, they were able to focus attention on individual features and suppress the holistic image of the entire composite.

Further research lends support to the interpretation that manipulations of global vs. local processing affect face recognition by influencing the extent to which perceivers are able to make use of the configural vs. feature information present in a face. For example, work by Martin and Macrae (2010) shows that individual differences in “global precedence” (i.e., the tendency to process Navon stimuli in a global manner) predicted the magnitude of the face inversion effect (FIE; that is, the decrease in face recognition accuracy when target faces are presented upside-down at test), with participants who showed a stronger global precedence also producing a larger FIE.

Evidence for a link between global processing and superior face recognition is not limited to studies employing Navon manipulations. For example, Perfect et al. (2007) asked participants to judge new faces either in terms of a feature (detailed information) or their personality (holistic information) during the interval between studying and recognizing unrelated faces. This manipulation produced the same effects as using the visuo-spatial Navon task: that is, the induction to judge a holistic attribute (personality) resulted in superior face recognition compared to an induction to focus on a perceptual detail (facial feature).

Other work also suggests that manipulations that induce feature-based processing (e.g., providing verbal descriptions, Schooler & Engstler-Schooler, 1990; processing other-race faces, Fallshore & Schooler, 1995; Meissner & Brigham, 2001; describing cars, Westerman & Larsen, 1997) undermine face recognition performance. Finally, N. A. Wyer et al. (2010) manipulated psychological distance by asking participants to think about an event in the near vs. far future during the interval between encountering a confederate and being asked to identify them from an array of photographs and found that performance was superior in the distant future conditions. Such manipulations of psychological distance have been commonly used to induce low- vs. high-level construal (Liberman & Trope, 2014; Trope & Liberman, 2010) which are, in turn, associated with relatively detail-based vs. holistic processing. In N. A. Wyer et al.’s (2010) studies, this manipulation then influenced face recognition accuracy, with greater distance (associated with high-level construal and holistic processing) leading to better recognition and greater proximity (associated with low-level construal and detail-based processing) being associated with poorer performance.

In a related line of work, Schwartz and Yovel (2016, 2019a, 2019b) have argued (and provided evidence) for the notion that face learning is improved when faces are treated in terms of their conceptual properties (e.g., the name or identity of the person shown) rather than as mere perceptual stimuli. In their studies, participants who are exposed to a single view of a face outperform those who are shown multiple views from different angles, *if* that single view was paired with a name (giving it an identity). Intriguingly, this phenomenon appears not to be driven by differences in processing style or elaboration.

## Recollecting Behaviors

While the distinction between holistic and detail-oriented processing styles has received considerable attention within the literature on face recognition, the question of how those styles influence memory for other types of information has received scant attention. Nonetheless, memory for social events is likely to include not only what others look like but how they behave, and what that behavior means for their personalities, moods, and goals. The literature on person memory has focused primarily on the extent to which specific behavioral details are retained in memory after an overall impression has been formed on the basis of those details (see Hamilton et al., 1980; R. S. Wyer et al., 1984). Studies into retrieval processes have been limited to testing hypotheses about associative links among behavioral episodes and between such episodes and the abstract personality traits that they reflect.

Thus, research into person memory has paid little attention to the role of processing styles in determining what is eventually recalled. Although evidence suggests that the capacity for systematic (vs. heuristic) processing influences the nature of recall (e.g., Bargh & Thein, 1985), this distinction bears more directly on depth of processing rather than style of processing. That is, experiments examining memory for behavioral information have reliably demonstrated that when perceivers elaborate on or reason about a target’s behavior (e.g., as when reconciling an apparently inconsistent behavior with the rest of one’s knowledge about the target; see Srull & Wyer, 1989 for a review), the ability to later recall that behavior is enhanced. In contrast, research investigating whether and how global or local processing styles affect behavioral memory is all but non-existent (cf. N. A. Wyer et al., 2010).

## Reconstructing the Background/Context

Social events typically include background objects and contextual information, but there is little research on memory for these within social contexts. There is, of course, reason to suspect that memory for objects and other contextual information might be generally poor when that information is encountered in the context of a social interaction. The salience of social behavior is likely to dominate attention (Wentura et al., 2000) such that other information is poorly encoded. Yet, there may be conditions in which such background details do attract attention and may be retrieved later. Evidence from cross-cultural studies (e.g., Miyamoto et al., 2006; see also Amer et al., 2017) suggests that individuals from cultures with a more holistic processing style (e.g., East Asians) are more likely to recognize background contexts (vs. focal objects) from a visual scene than are those with from a culture with a more detail-oriented processing style (e.g., Europeans or North Americans). In contrast, holistic processing is associated with inferior recognition of specific objects, particularly when those objects are presented against novel backgrounds (see Chua et al., 2005). Thus, individual differences in the tendency for holistic processing appear to predict the ability to accurately recognize contextual information (such as background information in a complex scene).

Cultural influences notwithstanding, evidence suggests that details of objects encountered within dynamic scenes (i.e., not static images—see Bainbridge et al., 2019 for evidence of object memory from static scenes) are scarcely encoded at all (Hirose et al., 2010). The phenomenon of “change blindness” (Simons & Rensink, 2005; Varakin et al., 2007) reflects this—although perceivers may detect that an object is present within a dynamic scene, they often fail to recognize when that specific object is replaced with a different one. Thus, under most circumstances, background objects may be less likely to afford multiple levels of encoding (i.e., of detailed or holistic information) in the same way that human faces or social behavior does. That is, when one encounters an object, we may not encode it in a great amount of detail under standard conditions. For example, imagine walking into a friend’s kitchen and seeing a blue coffee mug with some writing on it on the counter. Under standard conditions, you are likely to encode that object as merely “mug” or perhaps “blue mug.” Unless you carefully inspect the mug (e.g., because you use it yourself, or because your friend asks you to bring her “the blue mug with ‘a yawn is a silent scream for coffee” printed on it’ to differentiate it from available coffee mugs) you are unlikely to encode its finer details.

## When Might Construal Level Influence Remembering?

In formulating hypotheses about construal level’s likely effects on recognition and recall of different elements of a social event, it is not sufficient to speculate about the possible mechanisms through which such effects might emerge. We must also consider whether those mechanisms apply equally across distinct elements. That is, do variations in processing style at test, memory search, and reporting decisions have the same potential to affect different measures of memory, and memory for diverse event elements?

### When Information Contains Salient Detailed and Holistic Information

What does this analysis suggest about the effects of construal level on memory for various elements of a social event? We propose that a critical factor in determining whether construal level affects memory outcomes is the extent to which elements of an event are encoded at multiple levels. Some elements of a social encounter are readily amenable to encoding at multiple levels. For example, behavioral episodes are likely to convey both specific details (what was done) as well as abstracted inferences about goals, traits, and emotions (why it was done). Both levels of meaning are likely to be encoded when a behavior is observed (Klein et al., 1993; Winter & Uleman, 1984). Likewise, when encountering faces, perceivers encode both their features and holistic aspects (as evidenced by well-above-chance levels of recognition of face parts (Tanaka & Farah, 1993) and inverted faces (Yin, 1969). As such, these elements of a social stimulus are more likely to be influenced by shifts in construal which can alter the nature of the memory that is reported. However, as noted above, there is little evidence from which to speculate that objects and other aspects of the physical environment are routinely encoded at multiple levels. Thus, the extent to which memory for those elements might be affected by construal level at retrieval is less certain.

### The Role of Personal Involvement

One aspect that has not yet been discussed, but which is relevant to the research presented here, is the extent to which one is personally involved in the target event. In the course of our daily lives, we not only participate in social events but also observe those in which we are not personally involved as they unfold before us. In the case that we are merely observers, personal distance from the event may vary from close (as when we notice a couple having an argument at the next table at a restaurant) to remote (as when we watch television news coverage of something happening far away).

Personal involvement may moderate the effects of construal on event memory in two potential ways. First, high levels of personal involvement may invoke psychological proximity and thus promote low-level construal directly. In this case, we might expect low-level construal manipulations to have little effect (as the detail-oriented processing style that they are expected to induce should already be in operation). In contrast, low levels of personal involvement may entail greater psychological distance and thereby high-level construal. In that case, we may find that high-level construal manipulations have little effect (as the holistic processing style that they induce should already be in use). In other words, the effects of manipulating construal may be asymmetric, with larger effects observed when the manipulate contravenes the default processing style.

A second way in which personal involvement may impact on the effects of construal level is by altering the extent to which information is encoded in terms of both its holistic and detailed aspects. When personal involvement is low, perceivers may encode information only superficially, and may come away with only the gist of what happened during an event. In this case, manipulations that encourage detail-oriented processing are likely to have little effect on retrieval because event details have not been stored in memory in the first place. In contrast, high levels of personal involvement are likely to entail greater attention to all aspects of an event, including both detailed and holistic elements. If so, we should expect that the effects of construal level should be particularly strong when personal involvement is high, but weak or non-existent when personal involvement is low. Indeed, in other domains (e.g., the cognitive interview; see Kohnken, Milne, Memon, & Bull (1999)), manipulations to improve event memory have proven to be most effective when personal involvement is high, presumably because there is a richer memory representation that can be accessed in more or less effective ways (as opposed to events that receive little attention, where memory is likely to be poor regardless of the retrieval conditions).

## Summary: Formulating Predictions About Construal Effects on Remembering

In summary, then, while the influence of construal level on how people attend to, evaluate, and encode information is relatively well understood, little is known about how construal might alter how people retrieve information once it has been stored in memory. We aim to investigate the effects of construal level on how people remember events—an outcome that has been largely neglected in the plethora of research stemming from CLT (recent work linking construal level with working memory notwithstanding, Hadar et al., 2019). In particular, because of the uniquely multifaceted nature of social events, we focus on testing the hypothesis that construal level affects one’s retrieval of various aspects of one’s memory for a social encounter in distinct ways. We focused our investigation primarily on memory for visual and behavioral information, largely because these mapped onto both the expertise of the research team and because the evidence base relating to face memory and person memory is more extensive than other aspects that might also be considered (e.g., memory for sounds, or memory for the temporal order of events):

1. *Faces*: Work on face processing (N. A. Wyer, Hollins, & Pahl, 2015; N. A. Wyer, Hollins, Pahl, & Roper, 2015) has demonstrated that manipulations producing high-level construal encourage holistic processing (and hence greater accuracy) during face recognition tasks. Thus, we expect that high-level (relative to low-level) construal will be associated with more accurate recognition of faces encountered during social events.
2. *Free Recall of Behavior*: Prior research investigating memory for others’ behavior suggests that when an unfamiliar target is encountered, specific behaviors are encoded along with the trait implications of those behaviors (Hamilton et al., 1980). Thus, when prompted to recall information about a social encounter, perceivers should have access to both details of a target’s behavior and the meaning (e.g., personality trait inferences) ascribed to that behavior. We therefore expect that construal level will guide free recall of behaviors observed during social events, such that high-level construal will encourage retrieval of meaning-related information (e.g., personality trait inferences) whereas low-level construal will encourage retrieval of behavioral details.
3. *Cued Recall*: However, direct questions about the factual elements of a social event may not allow for flexibility to retrieve meaning versus detail from the event. We would therefore *not* expect to observe that construal level influences performance on closed-ended tests of memory for the event.
4. *Background*: We do not expect the memory advantage produced by high-level (relative to low-level) construal to generalize to object or background/contexts that are encountered in social interactions. We speculate that background information will be less focal and hence less likely to be encoded at multiple levels, making it less amenable to construal effects at retrieval.
5. *Physical Presence*: As alluded to above, we posit that greater personal involvement in an event is likely to result in richer memory representations. In the current work, the need to maintain experimental control meant that participants were typically not directly involved in the social encounters that served as memory targets. However, there was variation in the extent to which participants were physically present when the events occurred, and we use physical presence as a (albeit imperfect) proxy of personal involvement.

It is worth highlighting the point that we *do not* predict an *overall advantage* for high-level vs. low-level construal. Such generic superiority might be easily attributable to differences in motivation created by one or the other construal level, or by a general shift in retrieval strategy that improves memory across the board. Instead, we have identified specific elements of memory that, theoretically, should be susceptible to construal level influences, and other elements that are less likely to be affected.

## Early Evidence

As noted earlier, there is little research on how processing style influences memory for different aspects of an event. Earlier research from our own team (N. A. Wyer et al., 2010) represents the single exception. In these studies, participants encountered a confederate prior to an experimental task that independently manipulated psychological distance. Following the manipulation, participants’ memory for different aspects of the initial encounter was assessed. In both studies, participants in a distant-future condition were significantly more likely to be able to pick the confederate out of a line-up. One of the studies also required participants to give a verbal description of the initial encounter with the confederate which was coded for the nature of the descriptions used. Distant-future-focused participants were more likely to describe the meaning of the event (what the confederate wanted, the confederate’s personality) while near-future-focused participants were more likely to describe the specific sequence of events (what happened and what was said).

Taken together, these two studies suggest that construal level (manipulated in terms of temporal distance) can influence different aspects of event memory. However, because of the differences between the methodologies of those two initial studies, direct comparisons and hence theoretical conclusions are difficult. What we report in the next section is a series of closely interconnected studies which will allow greater insight into the generality of the construal effect in memory, and a deeper understanding of the theoretical mechanism underpinning these effects.

Beyond our interest in providing a rigorous test of how construal level affects the multifaceted and complex nature of how people remember social events, we are also motivated by our wish to fully report the results of a nearly 4-year research project. We embrace recent calls to resist publication bias (Cumming, 2014) and the difficulties it yields in establishing a reliable and replicable scientific record. In reporting our project in the following, we include every experiment regardless of the statistical significance of the effects. Some of these experiments were under-powered. Some of them were discontinued after preliminary analyses suggested that our hypotheses were not supported. Thus, the results of individual experiments often do not provide meaningful results. Our aim is to take a broader view of this line of work to provide a more rigorous test of our hypotheses, as well as more accurate estimates of effect sizes for those differences that do emerge.

## The Present Analysis

We undertook an extended program of research involving a total of 15 experiments which we summarize here.^2^ Following the model of Tuk et al. (2015), we used internal meta-analysis to give us maximum power to estimate the size of construal effects on memory. A variety of research paradigms were used over the course of the project (see Table 1), including laboratory, field, and on-line methodologies that required participants to recall aspects of different types of events (including those witnessed within the context of the experiment as well as those encountered independently of the experimental context).

**Table 1.** Summary of Experiments With Key Manipulations and Measures.

Exp.	*N*	Manipulation	Time of measure in relation to event	Type of event	Setting	Primary dependent variable(s)	Other measures
1^a^	90	Near future vs. Control vs. Distant future	Minutes after	Confederate encounter	Field	Face recognition	Self-reported global vs. detailed thoughts during manipulation task
2^a^	72	Near future vs. Distant future	Minutes after	Confederate encounter	Lab	Face recognition Narrative free recall	Number of categories formed during planning task
3	104	Near future vs. Distant future	Minutes after	Confederate encounter	Lab	Face recognition Background (Object/scene) recognition	Gestalt Completion Test, Snowy Pictures Task, Categorization, Analogical reasoning
4	145	How vs. Why	Minutes after	Confederate encounter	Lab	Face recognition Background (Object/scene) recognition	Gestalt Completion Test, Snowy Pictures Task, Categorization, Analogical reasoning
5	59	How vs. Why	Minutes after	Confederate encounter	Lab	Background (object/scene) recognition Narrative free recall	Gestalt Completion Test, Snowy Pictures Task, Categorization, Analogical reasoning
6	130	Near future vs. Distant future	Minutes after	Video	Online	Face recognition Closed-ended questions	
7	51	How vs. Why	Months after	Public Event (Royal Wedding)	Field	Face recognition Closed-ended questions	
8	100	How vs. Why	Months after	Public Event (Royal Wedding)	Field	Face recognition	
9	60	How vs. Why	Months after	Public Event (Royal Wedding)	Field	Narrative free recall	
10	100	How vs. Why	Months after	Public Event (Royal Wedding)	Field	Inverted face recognition	
11	74	How vs Why	Minutes after	Video	Lab	Face recognition Closed-ended questions	
12	75	How vs Why	Minutes after	Video	Lab	Face recognition Closed-ended questions	
13	59	Near location vs. Control vs. Distant location	Minutes after	Video	Lab	Face recognition Closed-ended questions	
14	63	How vs. Control vs. Why	Months after	Public Event (Various Media)	Lab	Narrative free recall	
15	48	How vs. Why	Months after	Idiosyncratic/ Autobiographical	Lab	Narrative free recall	

a Experiments 1 and 2 are reported in full elsewhere (see N. A. Wyer et al., 2010).

Our prototypical study was an experiment in which participants witnessed an event (either staged by a confederate or as a video-recorded scene) in which one or more target persons carried out a variety of actions in a natural environment containing clearly visible objects in the background. Either before or after the event, participants were induced to adopt either high-level or low-level construal using a variety of manipulations, including temporal distance, spatial distance, and more direct manipulations of construal (e.g., explaining how vs. why an action might be carried out). All of the manipulations used were based on prior research stemming from Construal Level Theory (see Trope & Liberman, 2011 for a review). In experiments where a confederate was used, the confederate carried out a precisely scripted series of actions and made a series of statements in the presence of participants (but not specifically directed toward them in all but one study). In other experiments, participants watched a video-recorded interaction between two actresses. In both versions, the room where the action took place was arranged such that we could assess participants’ memory for the configuration of objects within it.

We also carried out a series of field experiments in which we manipulated construal prior to testing participants’ memory for an event that they reported having witnessed (specifically the wedding of Prince William and Kate Middleton in the UK). In addition, two laboratory studies required participants to recall details of an event that they had personally experienced in the months or years prior to the experiment. Finally, we carried out an on-line experiment using a video-recording of a burglary in which a man forcibly enters a room and removes a number of objects. This range of stimuli and methodologies allowed us to test the effect of construal level on event memory comprehensively, including a number of potential moderators. While we had no a priori predictions regarding these moderators (described below), we include them to provide a means for assessing the robustness of construal level effects on memory.

Each experiment included one or more of the following dependent measures: face recognition (selecting a face from a line-up); action memory (true/false judgments about things that did or did not happen during the event, or identification of which of two characters engaged in an action or made a statement); narrative description of the target event (coded for detail and meaning); object and scene memory (recognition from a line-up, and true/false judgments regarding room arrangement).

## Method

### Overview and Participants

The effects of construal level on memory for social encounters were tested in 15 experiments with a total of 1230 participants. Gender was recorded for 991 participants (27.1% male) and age was recorded for 900 participants (*M* = 24.1 years, *SD* = 9.89). Neither gender nor age had any effect on memory measures (Gender: meta-regression slope for % male = −.001, 95% CI = [−0.008, 0.006], *Z* = −0.288, *p* = .773; Age: meta-regression slope = .005, 95% CI = [−0.013, 0.023], *Z* = 0.541, *p* = .588) and hence will not be discussed further. Of the 15 experiments, one was conducted on-line, five were carried out as field experiments, and the remaining 9 took place in laboratory settings. Detailed reports of the methods and results of individual studies are available here: https://osf.io/usvgy/

### Predictors

A number of factors are potentially important in determining the effects of construal level on memory and are hence included in our analysis as moderators. Each of these is discussed in the following in turn. The classification of each study along each factor can be found in Table 1.

#### Construal level manipulation

Three types of construal manipulation were used, two of which involved manipulations of psychological distance. Temporal distance manipulations asked participants to consider an event in the near or distant future. Spatial distance manipulations asked participants to consider an event in a close or distant location. The third type of manipulation involved a “How/Why” task in which participants were asked a series of questions about how or why one would engage in different actions (e.g., maintaining good health; see Fujita, Trope, et al., 2006). Proximal times and locations, and “How” manipulations were expected to produce low-level construals, whereas distant times and locations, and “Why” manipulations were expected to produce high-level construals (Liberman & Trope, 2014; Trope & Liberman, 2010). See Supplemental Methodological Appendix for details.

#### Time from event to memory measure

One might expect that details would be lost from memory over time, and in some of the studies included here a matter of months elapsed between the event in question and the point at which memory was measured. In other studies, memory was assessed mere minutes after the event had occurred. We therefore include time as a factor.

#### Physical presence

As noted above, target events varied widely across the studies. Of primary interest for the present analysis is the extent to which participants were personally present in the events. In this regard, each study was coded for physical presence as “high” if participants were physically present at the event, and “low” if participants viewed the event on screen (either via the media or via recordings made specifically for this research project).

#### Setting

Participants’ engagement with and attention to the event and the dependent measures may be influenced by the context in which they took part. We classified experimental setting as either field, lab, or online to account for these influences.

### Outcomes

#### Memory target

Across the experiments included the analysis, we assessed memory in a number of different ways:

*Face recognition* was assessed in nine studies by presenting participants with an array of six faces (head and shoulders photographs), which included a person who they had seen during the target event. Participants were asked to identify which of the faces they had seen earlier, and their responses were scored as correct or incorrect.

We measured *narrative (free) recall of behavior* in four studies by asking participants to remember and write down what had happened during an event. These open-ended descriptions were coded by two independent raters for their inclusion of behavioral details vs. abstract inferences (see section on *Coding and Scoring* in the following).

We also measured memory using *closed-ended* (true/false or multiple-choice) questions about specific details of the event or more global inferences that could be drawn from the event. Closed-ended questions were scored as correct or incorrect, and an average score was computed.

Three experiments also assessed memory for *background* information (e.g., presence/absence of objects or appearance of the physical environment). See Supplemental Methodological Appendix for details.

Finally, some measures were included in only a single study (recognition of inverted [upside-down] faces, an action identification task where participants chose between concrete and abstract descriptions of the same action) and so are not included in the analysis.

### Other Variables

Across several experiments, we also collected measures of processing style, including the Snowy Pictures and Gestalt Completion tests (Ekstrom et al., 1976), and a category breadth task (similar to that used in Fujita, Trope, et al, 2006), as well as a general measure of cognitive ability (an analogical reasoning task). Our preliminary two studies (N. A. Wyer et al., 2010) produced measures of how the construal manipulation was processed (self-reported global and detailed thoughts in Study 1, and the number of categories formed during a planning task in Study 2). The results from these measures have been included in a recent meta-analysis (Soderberg et al., 2015) and will be reported only briefly here.

## Results

### Coding and Scoring

Measures of face recognition, and recognition of background (object and scene) information, involved participants’ identification of a previously seen stimulus from an array of four to eight alternatives (face arrays always included six options, object arrays included eight options, and scenes were presented with four alternate versions) and were scored as correct or incorrect. Face recognition tasks typically involved a single trial, and responses were scored as correct or incorrect. Object and scene recognition included multiple trials, and hence an average score was computed. See Supplemental Methodological Appendix for examples.

Narrative (open-ended) recall was coded by two independent coders in each study where it was recorded. In cases where participants responded orally (in field studies only), responses were transcribed before coding. Coders were instructed to score each narrative on the extent to which it contained inferences or abstract information (including traits, emotions, goals/intentions) and, independently, on the extent to which it contained details of the event (including descriptions of visual details, things that were said and done, the order in which things occurred). Both ratings were made on 10-point scales (1 = none of this kind of information, 10 = a great deal of this kind of information). Reliability was satisfactory across studies (α’s = .89 to .95, average α = .93), with discrepancies between coders being resolved by a third independent coder.

Closed-ended questions about the event were scored as correct or incorrect, based on which an average score for each question type was computed.

### Meta-Analytic Approach

It is important to note that, in many studies included in this analysis, multiple memory measures are included. In such cases, assumptions of independence are violated (because performance by the same participants on one outcome are likely to be correlated with performance on a second outcome). We therefore adopt a two-pronged approach to this analysis. First, to assess the overall effects of key manipulations, we combine all measures within a study following the procedures outlined by Cumming et al. (2012). Second, to assess the effects of construal level on specific outcomes, we carry out separate meta-analyses on each, followed up by between-group comparisons when there is significant heterogeneity in the overall effect on that outcome.

**Figure 1.**
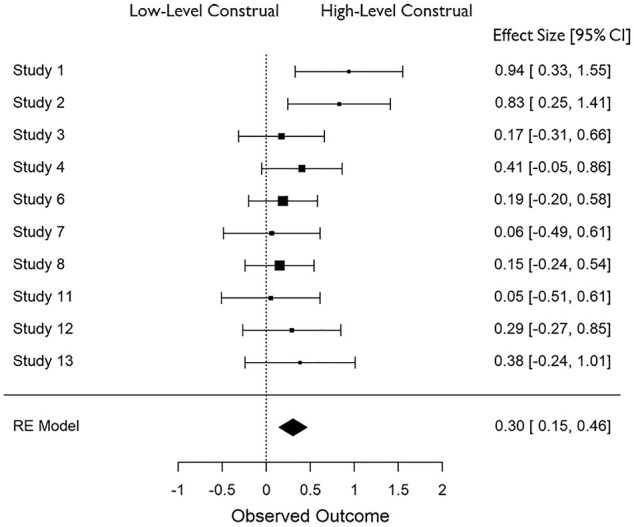
Accuracy in recognizing faces (*K* = 10). *Note.* CI = Confidence Interval.

**Figure 2.**
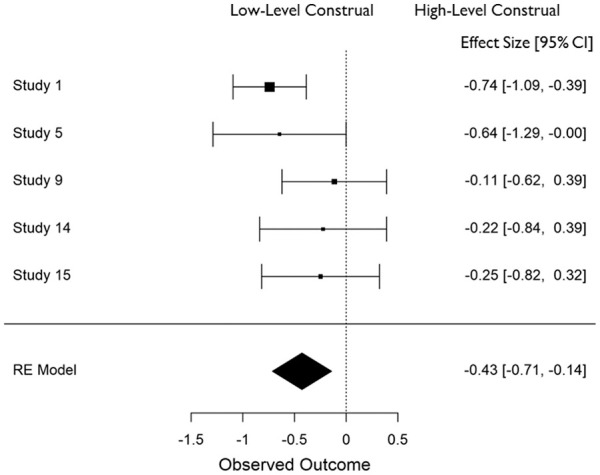
Use of detail in (open-ended) narrative recall (*K* = 5). *Note.* CI = Confidence Interval.

**Figure 3.**
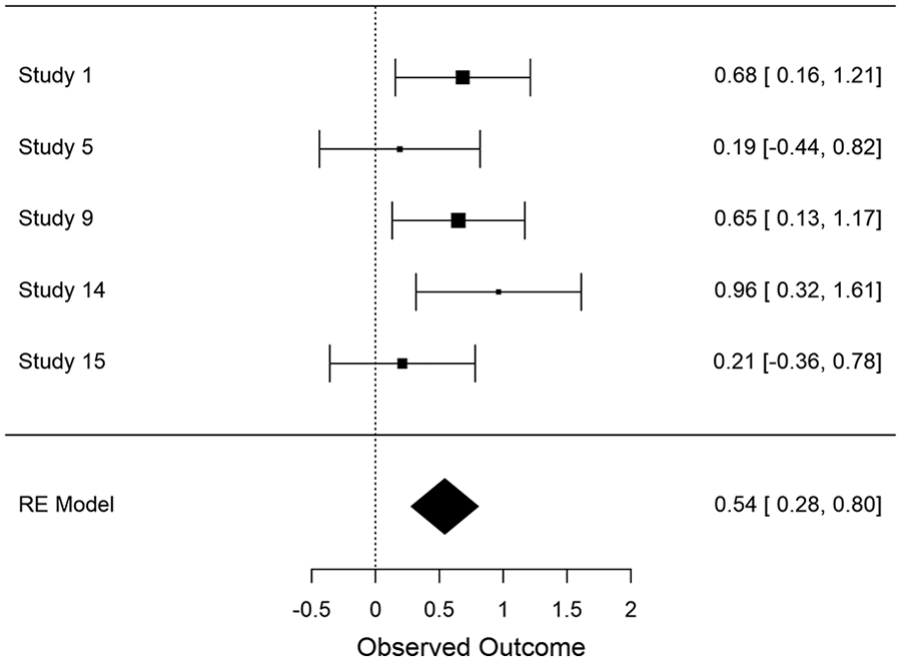
Use of global descriptions in (open ended) narrative recall (*K* = 5). *Note.* CI = Confidence Interval.

**Figure 4.**
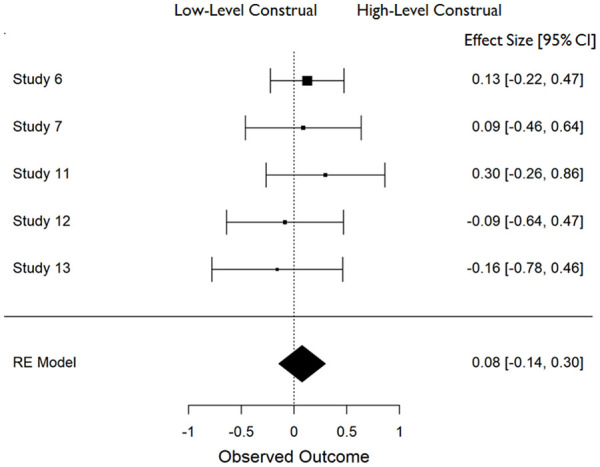
Accuracy in answering (closed-ended) questions about event details (*K* = 5). *Note.* CI = Confidence Interval.

**Figure 5.**
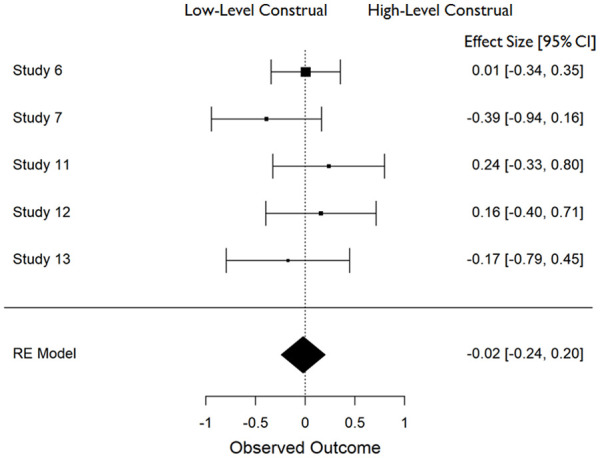
Accuracy in answering (closed ended) questions about global event information (*K* = 5). *Note.* CI = Confidence Interval.

**Figure 6.**
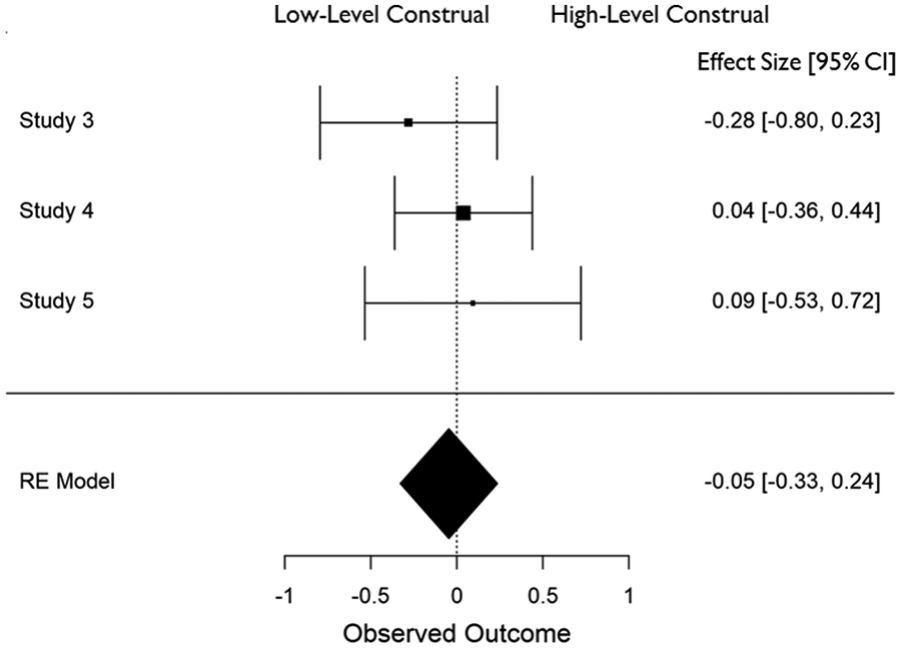
Accuracy in recognizing background objects (*K* = 3). *Note.* CI = Confidence Interval.

In all cases, effect sizes for the difference between high- and low-level construal conditions were converted to standardized mean differences (*d*), and aggregate data analyses were carried out in JASP (JASP Team, 2020) using a restricted maximum likelihood model of random effects. The distribution of effect sizes across measures can be seen in Figures 1 to 6.

Following current conventions in reporting the results of meta-analyses, we will focus primarily on comparisons of effect size rather than significance levels. We will also refrain from discussing effect sizes where those are based on a single study. Following recommendations by von Hippel (2015) we note that in many cases we have insufficient power (given the small number of studies) for tests of heterogeneity to be reliable; we are therefore guided by our theoretical questions in examining between-study variability. Finally, note that all effect sizes should be interpreted such that positive numbers reflect an advantage for high-level (over low-level) construal on upright face recognition or measures of memory for global features of the event, but an advantage for low-level (over high-level) construal on memory for detailed aspects of the event.

### Overall Effect of Construal on Memory

The average effect size for all memory outcomes (including face recognition, object/scene recognition, and both open-ended and closed-ended forms of behavioral recall) between Low-Level *vs.* High-Level Construal conditions was *d* = 0.107 (*SE* = .065), 95% CI = [−0.021, +0.235], *Z* = +1.645, *p* = .100. However, there was significant heterogeneity in effect sizes, *Q*(33) = 71.169, *p* < .001, *I*^2^ = 52.943. Our hypotheses suggest that larger effects should be observed for some aspects of memory (e.g., face recognition, recall of abstracted person information) while we expect no effect on other aspects (e.g., object and scene recognition) and even negative effects on others (e.g., recall of detailed person information). Thus, we carried out sub-group analyses on specific memory outcomes.

#### Effect of construal on face recognition

Construal level had a small-to-medium effect on face recognition accuracy, *d* = 0.304 (*SE* = .08), 95% CI = [+0.146, +0.462], *Z* = 3.767, *p* < .001 such that performance was higher in high-level construal conditions than in low-level construal conditions (see Figure 1). Notably, the effect was only present when participants had encountered the target very recently (*d* = 0.364) and not when the exposure to the target was more distant in time (*d* = 0.122). Furthermore, not all construal manipulations had equivalent effects: face recognition was improved by high-level construal mainly when it was manipulated via psychological distance (time: *d* = 0.438; space: *d* = 0.385) rather than using the how vs. why task (*d* = 0.201), as well as when participants were physically present at the event (*d* = 0.522) rather than watching a recording of the event (*d* = 0.179). Finally, the effect was larger when the study was carried out in person (field: *d* = 0.318; lab: *d* = 0.346) versus on-line (*d* = 0.191).

#### Effect of construal on coding of narrative (open-ended) recall

Open-ended narrative recall was coded for the amount of detail/concrete information and meaning/abstract information that was included. Construal level had a medium effect on the amount of detail included, *d* = −0.427 (*SE* = .146), 95% CI = [−0.713, −0.142], *Z* = −2.931, *p* = .003 such that more detail was generated in low-level than high-level construal conditions (see Figure 2). Notably, construal only impacted the recall of details when the event had occurred in the recent past (*d* = −0.718) but not in the more distant past (*d* = −0.187). Construal level had a medium effect on the amount of abstract information that was included, *d* = 0.542 (*SE* = .133), 95% CI = [+0.281, +0.804], *Z* = 4.070, *p* < .001 such that high-level construal gave rise to more abstract recall than did low-level construal (see Figure 3). In contrast to the presence of detail in participants’ open-ended descriptions, there was a larger impact of construal on global descriptions when the event occurred in the more distant past (*d* = 0.590) compared to when it was more recent (*d* = 0.467).

#### Effect of construal on closed-ended questions about behavior

Construal level had no effect on participants’ ability to correctly respond to closed-ended questions about the event they had witnessed. This was equally true of questions relating to event details, *d* = 0.077 (*SE* = .113), 95% CI = [−0.144, +0.298], *Z* = 0.682, *p* = .495, those relating to global aspects of the event, *d* = −0.02 (*SE* = .113), 95% CI = [−0.241, +0.201] *Z* = −0.176, *p* = .861 (see Figures 4 and 5).

#### Effect of construal on background (object/scene) recognition

Construal level also had no discernible effect on participants’ ability to accurately recognize objects and background scenes, *d* = −0.046 (*SE* = 0.144), 95% CI = [−0.328, +0.236] *Z* = −0.321, *p* = .748 (see Figure 6).

### Effectiveness of Construal Manipulations

Although we did not carry out specific manipulation checks in every study, those that we did obtain suggest that construal manipulations are generally very effective in producing processing differences in relation to the content they apply to. Effects of Construal Level on manipulation tasks were highly reliable, *d* = 0.68 (*SE* = .16), 95% CI = [+0.38, +0.99], *Z* = 4.40, *p* < .001.

### Effect of Construal on Other (Non-Memory) Measures

**Figure 7.**
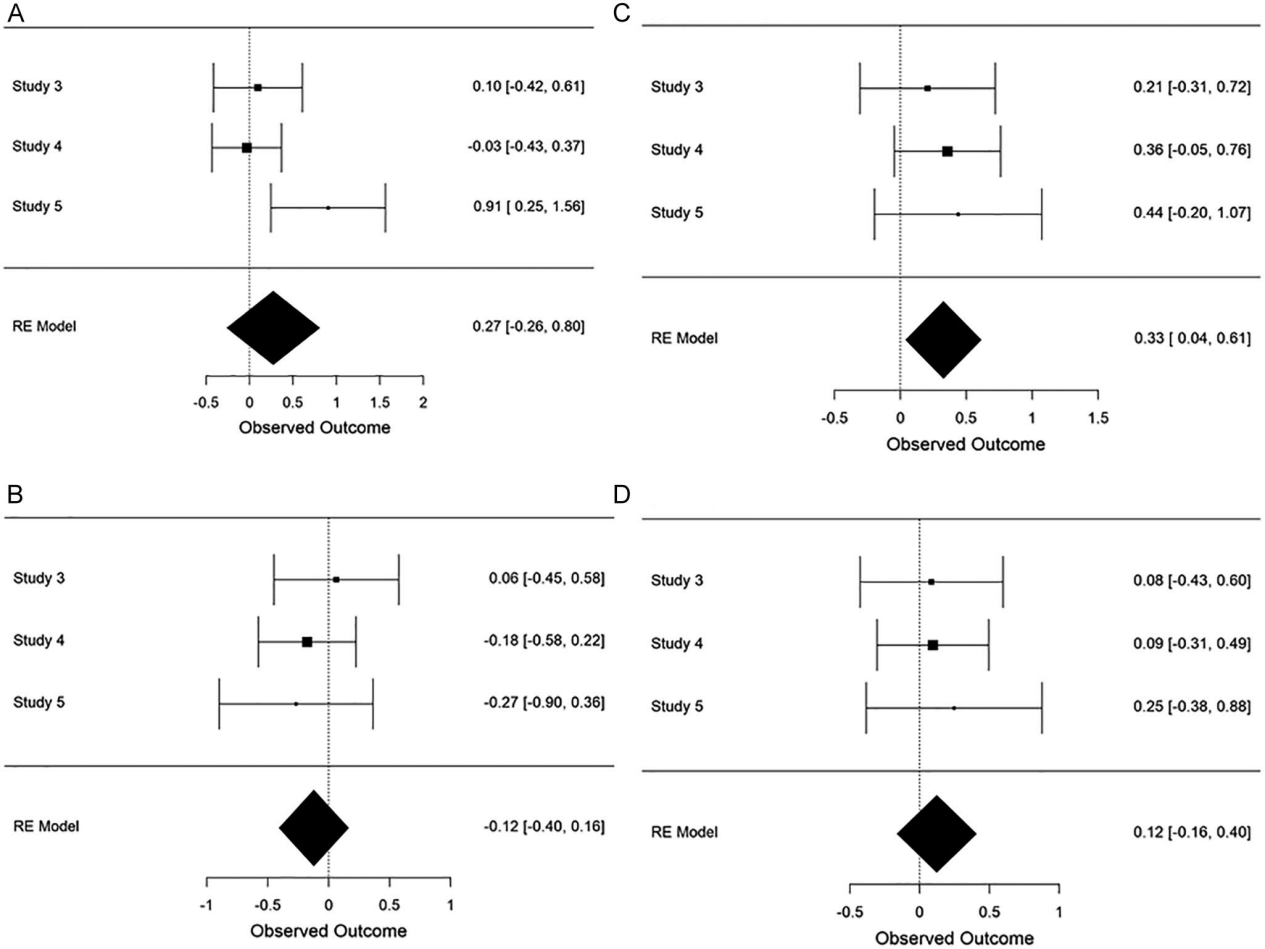
Performance on processing measures: (A) Analogies, (B) Categorization, (C) Gestalt completion test, and (D) Snowy pictures test.

While not the primary focus of this research, a number of studies included measures that were intended to detect differences in processing style, which may contribute to memory outcomes. An analysis of global processing measures reveals a small effect of construal level on processing outcomes, *d* = 0.126 (*SE* = .072), 95% CI = [−0.015, +0.268] *Z* = 1.751, *p* = .080 such that high-level construal led to superior performance on such measures. Notably, few effects reached significance in individual studies (see Figure 7), and only one measure (the Gestalt Completion Test) produced a reliable meta-analytic effect.

## General Discussion

Research based on construal level theory has revealed wide-ranging effects of construal level on a variety of outcomes, including judgments, evaluations, and decisions (for a review, see Liberman & Trope, 2014; Trope & Liberman, 2010). These effects have been posited to emanate from the influence of construal level on basic processing style—high-level construal is argued to induce holistic or global processing, whereas low-level construal is believed to give rise to detail-oriented or local processing. The present research was designed to investigate the extent to which construal level and, by extension, processing style affect memory for diverse aspects of social events. While previous research (N. A. Wyer et al., 2010) has suggested a link between psychological distance (a manipulation often linked to construal level) and social aspects of memory (face recognition and behavioral recall), no previous research had examined the effects of construal level on memory directly, or extended them to memory for background or non-focal aspects of the event (e.g., memory for the physical environment).

The present work provided this investigation, and resulted in a number of important findings. First, construal level was shown to have a robust effect on subsequent face recognition. High-level construal was consistently associated with more accurate face recognition when compared to low-level construal. Moreover, these effects were not limited to faces that had been seen in the recent past, but extended to a face encountered only once at least five months earlier (e.g., Studies 7 and 8).

Second, we demonstrated that construal level has a complex relationship with memory for behavioral details and inferences (or gist information). Although construal level had a significant effect on the content of participants’ narrative reconstructions of events, the nature of the effect differed across studies. When the target event was one that had occurred in the very recent past, construal level altered the likelihood that behavioral details emerged as part of participants’ recollections (with low-level construal promoting greater recall). In contrast, when the target event happened in the more distant past, construal level affected the likelihood of retrieving inferences about the event (with high-level construal eliciting greater levels of inference in participants’ recollections).

Finally, we found that the effects of construal level—at least in our studies—was restricted to memory for the focal social elements of the event, and had no discernible effect on recognition of background objects or other visual details that were present. One clear explanation for this finding is that social information was likely to attract more attention. Actors and confederates—unlike objects in the room—are dynamic and thus more inherently interesting, whereas the physical information simply formed the background context against which the actors were viewed. Second, social aspects of an event may inherently present a richer array of information, including both detailed and holistic elements. This may be particularly true when one is physically present during the target event, yielding the possibility that information is encoded at multiple levels (i.e., both its detailed and holistic aspects). Given our premise that construal level alters the way in which information is retrieved from memory, such effects are likely to occur only when there are multiple options for retrieval. In other words, if event details are not stored in memory, they cannot later be retrieved regardless of the cognitive process applied toward retrieval.

### Possible Routes Through Which Construal Influences Memory

Because of the established effects of construal level on processing style in general, we postulate three potential routes through which it may influence social memory: by inducing holistic vs. analytic processing, by altering the strategy with which people search their memory for information, and by affecting the evidential threshold that is applied when determining what “counts” as a valid memory (or an aspect of memory that is relevant to report). In other words, beyond direct influences on the overall processing style with which perceivers operate when remembering an event, construal level might also affect more deliberate memory strategies—both in terms of how people *search* their memory for information they’ve encountered, and also how people decide how to *retrieve and report* the information from that search.

Construal level has, in previous work, been found to directly influence processing style. High-level construal has been observed to improve performance on tasks that require gist extraction (e.g., Snowy Pictures and Gestalt Completion Tests, Ekstrom et al., 1976) while low-level construal is advantageous for tasks that require attention to details (e.g., Embedded Figures task, Witkin et al., 1971). Recently, we have established that construal level also affects the use of holistic processing in face perception (N. A. Wyer, Hollins, Pahl, & Roper, 2015) which also benefits face recognition (N. A. Wyer, Hollins, & Pahl, 2015). Yet, in the present work, we found little evidence that processing style was affected by our manipulations in ways that paralleled their effects on memory. These findings are discussed further in the following; however, it remains the case that—at least in the paradigms employed in this work—we found no support for the idea that construal’s effects on memory were mediated by processing style.

The other two possibilities—that construal level influences memory via its effects on search strategies or reporting decisions—are more plausible accounts for our findings. Both of these accounts assume that the contents of memory are unaffected by construal manipulations—only the retrieval and reporting from memory is impacted. In all but one of the studies reported here, the target event took place *before* construal level was induced. Thus, at the time that construal level was manipulated, participants should have stored an equivalent representation of the target event. We suggest that construal level influenced participants’ strategies for accessing those representations and/or for reporting them in response to memory probes.

In support of search strategies as a contributing factor, Eyal et al. (2011) reported that participants who adopted high-level construals were more likely to engage in schema-driven processing of information when forming judgments of others. An increased reliance on schemas may also affect memory such that individuals are biased to retrieve schema-consistent information (Bower et al., 1979; Shank & Abelson, 2013) and may neglect details that are irrelevant to or inconsistent with that schema (Rothbart et al., 1979). Similarly, research drawing on the fuzzy trace theory of memory (Brainerd & Reyna, 1990; Reyna & Brainerd, 1995) suggests that schema use promotes retrieval of gist information at the expense of verbatim details (Tuckey & Brewer, 2003). Thus, based on evidence that high-level construal promotes schema-based processing, participants in our studies who used high-level construal may have been more likely to recall general themes (or gist information) from an event at the expense of details (see Bartlett, 1932).

Alternatively, construal level may have influenced performance on memory measures by affecting decision processes involved in reporting the contents of one’s memory (see N. A. Wyer, Hollins, Pahl, & Roper, 2015). Such decision processes may play a role in both recognition and recall memory. When individuals are charged with reporting whether or not an event occurred or whether or not they have seen someone before (i.e., recognition), they must not only retrieve relevant information from a stored memory, but they must also compare the retrieved material to the target item to determine whether they match. Construal level may influence the weight given to relatively holistic vs. detailed information in making such a determination (N. A. Wyer, Hollins, Pahl, & Roper, 2015). In recall tasks, where one is required to generate a description of an event that has transpired, one must first employ some search strategy to retrieve information about the event (as described above), and then engage decision processes to determine whether material retrieved from memory is relevant to the task at hand. As noted above, construal level may influence the kind of information that is retrieved; however, even if the information retrieved is itself invariant, construal level may influence the weight given to different pieces of information (e.g., specific vs. general) from which one constructs their memory report.

Thus, we do not suggest that memory representations themselves were impacted by construal manipulations within the current studies. Conversely, over time, should the same construal level be in place when the event is recalled, the representation may change such that inferences are reinforced or that details are either retained or lost. In the domain of face recognition, this interpretation is compatible with what we understand about the effects of verbal description on face processing. Research by Schooler and others (Schooler, 2002; Schooler & Engstler-Schooler, 1990) on the “verbal overshadowing” effect has demonstrated that participants who view a face and are then asked to provide a verbal description of it are subsequently less successful at recognizing the face. Although various accounts have been proposed to explain this phenomenon (see Meissner & Brigham, 2001 for a review), most research suggests that the stored representation of face does not necessarily change. Rather, the act of providing a verbal description induces a relatively detail-oriented processing style, which inadvertently carries over to the face recognition task.

### Event Memory vs. Autobiographical Memory

Our focus here has been on how construal level influences the way that people recall events that they have witnessed. As noted at the outset, events—unlike photographs of faces or written descriptions of a person’s behavior—are complex and dynamic, containing both visual and semantic elements that change over time as the event unfolds. The literature on memory for observed social events has only scratched the surface when it comes to understanding such memories (cf R. S. Wyer et al., 2002 for a review of research on memory as narrative), In contrast, research into memory for lived experiences—i.e., autobiographical memory—has received comparatively extensive empirical attention, and so it is worth commenting on how this work can be situated within social memory (including autobiographical memory) more broadly.

The line of autobiographical memory research that is perhaps most closely aligned to the present work is that which has explored the relationship between visual perspective and the psychological distance of autobiographical memories. Work by Libby and colleagues (see Libby & Eibach, 2011 for a review) has established a close correspondence between the extent to which people feel psychologically close to events in their past and their use of a first-person perspective in imagining those events. When people conjure mental images of their past using a first-person perspective, they associate those events with more intensity, with more concrete detail, and as of greater relevance to the current self. In contrast, adopting a third-person perspective when recalling past events is associated with greater feelings of detachment and abstraction. There, as in our work, greater psychological distance and greater levels of visual and conceptual abstraction are linked.

The association between psychological distance and the detail with which autobiographical events are imagined has broad implications. For example, witnesses to criminal acts are often encouraged to visually recreate the context in which the events occurred, encouraging a first-person perspective (Geiselman et al., 1986). Such an approach is believed to be more likely to yield valuable details about the crime. Yet, to the extent that such instructions induce psychological proximity (rather than distance), they may constrain the information that is likely to emerge—while witnesses may be better able to report the specific actions undertaken by criminal suspects, their ability to recognize those suspects (e.g., from photos or in a line-up) may suffer. Further research might fruitfully explore whether the same memory representation might be accessed multiple times, under alternating conditions of psychological distance and proximity, to maximize the accuracy of both types of memory (see Butler et al., 2016).

### Construal and Processing Measures

One final aspect of the present work that bears mention is the finding, across the first three studies, that construal level was not consistently associated with any of the three measures of processing style (the Gestalt completion task, the snowy pictures task, and the category inclusion measure), and yielded only a weak overall effect on processing measures. These results are surprising in that they represent a divergence from prior research in which manipulations that are believed to alter construal level (e.g., power, as in Smith & Trope, 2006) have led to performance differences on these tasks. What is particularly striking is that construal level failed to influence these tasks in the same studies that it did affect memory measures. Notably, our experiments differed from previous studies in which construal level has been found to influence processing style, in that they included a larger set of measures (multiple processing measures in addition to the main memory measures). The relatively “busy” experimental sessions may have diluted the effects of construal manipulations on processing, which was always measured after the memory tasks that were our main focus. It also bears consideration, however, that effects of construal level on memory may be independent of their effects on basic perceptual and conceptual processing, and that there may be boundary conditions on the relationship between construal level and these (perceptual and conceptual) measures of processing style. Further study is required to identify the conditions under which these relationships are and are not likely to emerge.

### Conclusion

The present research demonstrates that construal level influences multiple aspects of memory for social encounters, while also highlighting some potential limitations to the effects of construal level. The studies reported here constitute the first known attempt to examine memory for multiple elements of the same event, and memory for events in both the recent and more distant past, within the same program of work. Thus, this research lays the groundwork for future investigations of the complexity of event memory and the factors that influence it.

## Supplemental Material

sj-docx-1-psp-10.1177_01461672211038188 – Supplemental material for Remembering Social Events: A Construal Level Approach Click here for additional data file. Supplemental material, sj-docx-1-psp-10.1177_01461672211038188 for Remembering Social Events: A Construal Level Approach by Natalie A. Wyer, Timothy J. Hollins and Sabine Pahl in Personality and Social Psychology Bulletin

## Notes

1 As noted previously, there are extensive, though separate, literatures on memory for faces, behaviors, objects, and scenes. For purposes of brevity, we restrict the present review to work that is particularly relevant to how processing style might affect each of these elements, particularly in the context of social events.

2 We have carried out several further experiments investigating construal level effects on face recognition and face processing. We do not include these experiments in the present analysis because they did not expose participants to a social event (instead, they employed standard face inversion or facial composite paradigms that involved presentation and test of multiple serially-presented face stimuli). Construal manipulations produced effects on face processing and recognition, with effect sizes that are comparable to those reported here. Details of these experiments are published elsewhere (N. A. Wyer et al., 2012; N. A. Wyer, Hollins, & Pahl, 2015; N. A. Wyer, Hollins, Pahl, & Roper, 2015), and details are available from the authors.
