# Supplementary material for: Remembering Social Events: A Construal Level Approach
Source: Pers Soc Psychol Bull. 2021 Aug 18;48(8):1238–54. doi: 10.1177/01461672211038188 (PMC9245159; doi:10.1177/01461672211038188)

Methodological Appendix

*Events*

Various types of events were used across experiments:

*Confederate Encounters:* Three types of confederate encounters were used, each of which followed a pre-arranged and rehearsed script. Details of two of these are provided elsewhere (Wyer et al., 2010 Experiments 1-2). In Experiments 3-5, participants arrived (individually or in small groups) at a laboratory room and were greeted by a female confederate who appeared to have been working in the room. The room was arranged with various items of furniture and other objects. The confederate informed participants that she was just finishing but that they could come in to wait for their experimenter to arrive. After participants were seated, the confederate proceeded to put her belongings away and to re-arrange items in such a way as to draw attention to various objects in the room. She then staged a phone call to someone in which she appeared to be distressed about being late. As she concluded the call, the experimenter arrived. They had a brief exchange, after which the confederate exited the room and participants went on to complete the rest of the experiment.

*Video:* Two videotaped events were used. In Experiment 6, participants viewed a video showing a man breaking into a house through a window, searching the surface and drawers of a desk, and removing various items. In Experiments 11-13, participants viewed a video showing two women talking about a third person whilst carrying out various actions (pouring coffee, looking through a magazine, etc). This scene was set in a room where specific objects and furniture were in constant view.

*Public/Media Events*: In Experiments 7-10, participants were recruited on the basis that they reported having watched television coverage of the ‘Royal Wedding’ (between the UK’s Prince William and Katherine Middleton) which had taken place approximately four months earlier. In Experiment 14, participants were given a list of widely-seen video-recorded events (including public events (e.g., the torch being lit at the London Olympics opening ceremony), YouTube videos (e.g., ‘Charlie bit me’) and scenes from well-known films (e.g., ‘Dark Knight Rises’ – Batman in the ‘Pit’). After completing a construal manipulation, they were given a list of the 5 events they had rated most familiar and instructed to write a description of each.

*Idiosyncratic*/*Autobiographical Events:* In Experiment 15, participants were given a number of cues (e.g., mountains, Halloween, childhood friend) and asked to generate a memory of something that had happened between 2 and 5 years in the past. After completing a construal manipulation, they were instructed to write a description of each memory they had generated.

*Construal Manipulations*

*Temporal Distance*: Manipulations of temporal distance were used in Experiments 1-3 and Experiment 6. Experiments 1 and 2 are described in full elsewhere (Wyer et al., 2010). Experiment 3 used the same manipulation as Experiment 2 (see also Freitas et al, 2004) which required participants to sort items into groups for use in the near or distant future. Experiment 6 required participants to write a paragraph describing what they would be doing the upcoming weekend (near future) or the weekend before Christmas (distant future)

*Spatial Distance:* Experiment 13 included a manipulation of spatial distance, in which participants were informed that the scene they viewed on video took place in a nearby location (at their own university) or in a distant location (a university in another part of the country).

*How/Why*: The remaining experiments manipulated construal level using a variant of the How vs. Why task described in Fujita et al (2006). Participants were given an action (e.g., Maintaining good health) and were asked to describe either *how* they would carry out that action, or *why* they would carry out that action. After their initial response, they were then asked how or why they would do the thing they described in their response. For example, in the *how* condition, if they responded ‘by exercising’ they would then be asked how they would exercise); in the *why* condition, if they responded ‘because I want to feel good’ they would then be asked ‘why do you want to feel good’). This process was repeated two more times (always becoming more detailed in the ‘how’ condition or more abstract in the ‘why’ condition).

*Memory Measures*

*Face Recognition*: Face recognition was measured using photographic arrays in which the target face was presented along with five alternatives who were superficially similar in appearance (matched for age, race, gender, hair length and color) and without any distinguishing features. An example can be seen in Figure A1.

*Figure A1.*


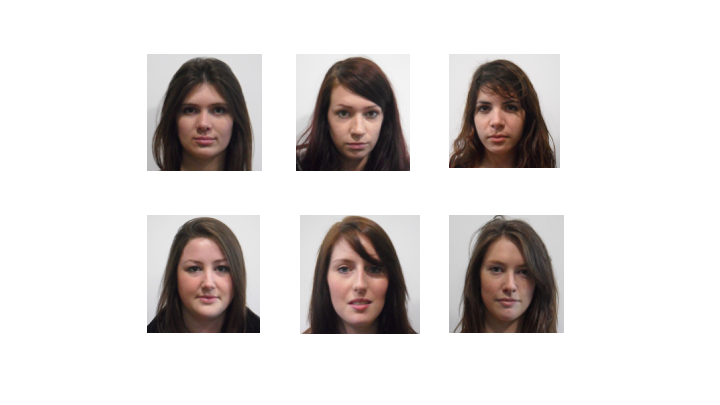


*Object recognition*: Object recognition was measured in the same way as face recognition. For each target object, seven similar distractor objects were included in the array. An example can be seen in Figure A2.

*Figure A2*


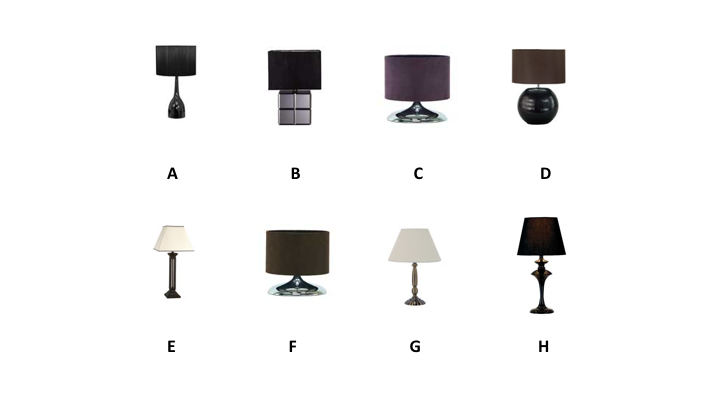


*Scene recognition*: Scene recognition measures provided participants with four alternative views of different parts of the room in which the event occurred, one of which corresponded to the view they actually saw. An example can be seen in Figure A3.

*Figure A3.*


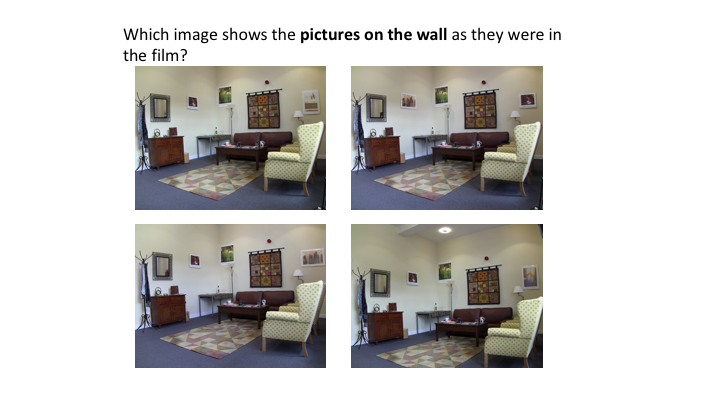

Supplement: sj-docx-1-psp-10.1177_01461672211038188 – Supplemental material for Remembering Social Events: A Construal Level Approach [file sj-docx-1-psp-10.1177_01461672211038188.docx]
